# Signal-sequence induced conformational changes in the signal recognition particle

**DOI:** 10.1038/ncomms8163

**Published:** 2015-06-08

**Authors:** Tobias Hainzl, A. Elisabeth Sauer-Eriksson

**Affiliations:** 1 Department of Chemistry, Umeå University, Umeå SE-901 87, Sweden

## Abstract

Co-translational protein targeting is an essential, evolutionarily conserved pathway for delivering nascent proteins to the proper cellular membrane. In this pathway, the signal recognition particle (SRP) first recognizes the N-terminal signal sequence of nascent proteins and subsequently interacts with the SRP receptor. For this, signal sequence binding in the SRP54 M domain must be effectively communicated to the SRP54 NG domain that interacts with the receptor. Here we present the 2.9 Å crystal structure of unbound- and signal sequence bound SRP forms, both present in the asymmetric unit. The structures provide evidence for a coupled binding and folding mechanism in which signal sequence binding induces the concerted folding of the GM linker helix, the finger loop, and the C-terminal alpha helix αM6. This mechanism allows for a high degree of structural adaptability of the binding site and suggests how signal sequence binding in the M domain is coupled to repositioning of the NG domain.

## Abstract (web summary)

The signal recognition particle plays a key role in the co-translational protein targeting of membrane and secretory proteins. Here the authors report a crystal structure of the ternary SRP complex in signal sequence bound and unbound forms, providing insight into how signal sequence binding is coupled to SRP receptor interaction.

## Graphical abstract

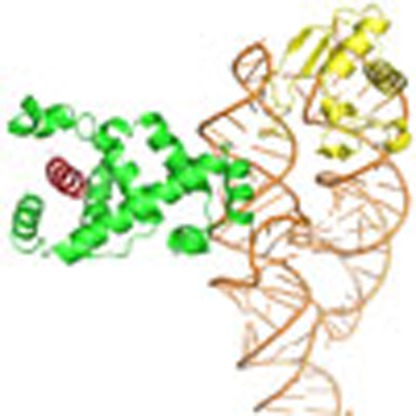

The signal recognition particle (SRP) co-translationally targets proteins to the endoplasmic reticulum in eurkaryotes or to the plasma membrane in prokaryotes. As the initiating step, SRP binds to the N-terminal signal sequence of nascent secretory or membrane proteins as they emerge from the ribosome. The SRP–ribosome nascent chain complex (RNC) is then targeted to the membrane through a GTP-dependent interaction with the SRP receptor (SR). The signal sequence is released from SRP and inserted into the translocon channel. Finally, GTP hydrolysis triggers the dissociation of SRP from SR, and SRP can start another cycle of protein targeting (for review see ref 1, 2).

SRP composition varies in the three domains of life. However, the evolutionary conserved SRP core only comprises the SRP54 protein (termed Ffh in bacteria) bound to the coaxial stacked helices 5 and 8 of SRP RNA. It is this SRP core that carries out the key functions of signal sequence recognition and SR interaction.

SRP54 comprises an N-terminal NG domain and a C-terminal methionine-rich M domain. The NG domain contains the GTPase activity and interacts with SR^3^,^4^. The M domain anchors SRP54 onto the SRP RNA and contains the signal sequence binding site^5^,^6^,^7^,^8^. The NG and M domains are connected by a long and flexible GM linker that allows for rearrangements of the relative positions of the M and NG domains^6^,^9^,^10^,^11^,^12^,^13^,^14^. Biochemical studies show that the binding of a signal sequence to the M domain accelerates GTP-independent interaction between the NG domain and SR at the helix 8 tetraloop^15^,^16^. This ‘early' complex undergoes a GTP-dependent structural change that results in a ‘closed' and stable complex. Large-scale rearrangement of the closed complex to helix 5 leads to GTPase activation and signal sequence release^11^,^16^,^17^,^18^. The essential SRP RNA functions as a scaffold to mediate the rearrangements; it is also required for efficient GTPase activation^11^,^17^.

SRP-dependent N-terminal signal sequences are highly diverse in amino-acid composition and length, but they all contain a core of at least eight consecutive hydrophobic amino acids that acts as the major determinant for recognition by SRP^19^,^20^. In addition, the N-terminus of a signal sequence typically contains positively charged residues with an as yet unknown function.

Two crystal structures of archaeal SRP54-signal sequence fusion proteins have provided the first detailed views of the M domain-signal sequence interaction. The M domain contains five amphipathic α-helices (αM1-5) and includes a ‘finger loop' between αM1 and αM2 (ref. 7). Different orientations of the signal sequence are seen in the crystal structures. In *Sulfolobus solfataricus* SRP54 complexed with the signal sequence of yeast dipeptidyl aminopeptidase B (DPAP-B)^5^, the signal sequence binds into a groove formed by αM1, αM2 and αM5; it is positioned roughly antiparallel to αM5. For the complex of *Methanococcus jannaschii* SRP54 and a signal sequence mimic^6^, the signal sequence is positioned perpendicular to αM5. There it is bound in a shallow groove formed by the αM1, αM5 and GM linker helix (Supplementary Fig. 1). A major drawback of both crystallographic models is that the SRP54 M domain is complexed with a signal sequence from a different SRP54 protein in the asymmetric unit. This results in a non-physiological SRP54 dimerization, both in solution and in the crystals^5^,^6^.

Communication between the M and NG domains is critical for SRP function. During protein targeting, the binding of a signal sequence in the M domain must be effectively transmitted to the NG domain to accelerate its interaction with SR. The molecular mechanism by which the signal sequence stimulates NG domain–SR complex assembly is currently not understood. To address this, we performed structural studies on *M. jannaschii* SRP and its interaction with signal sequences. Here we report the crystal structure of a monomeric SRP, including the full-length (fl) M domain in complex with a signal sequence. This structure shows how signal sequence binding in the M domain is directly linked to NG domain repositioning via an induced helix formation of the GM linker.

## Results

### The full-length SRP54-signal sequence fusion is monomeric

**Figure 1:**
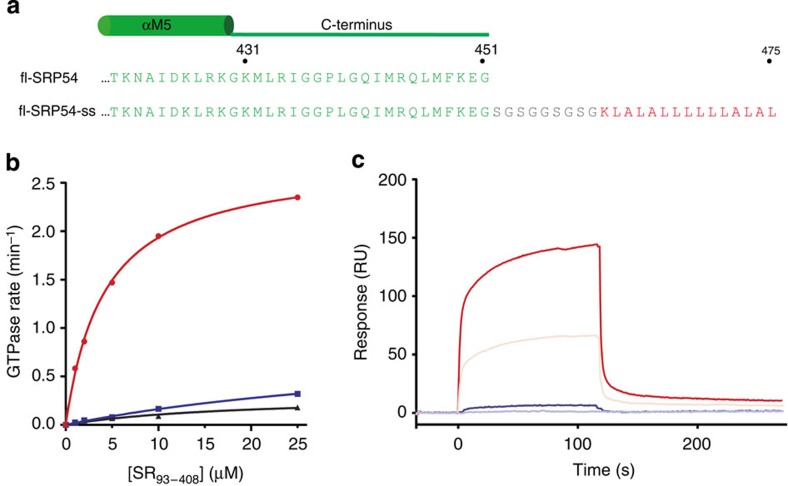
The fused signal sequence promotes *M. jannaschii* SRP–SR interaction. (**a**) C-terminal amino-acid sequence of *M. jannaschii* fl-SRP54 and fl-SRP54-ss. The colour code is as follows: M domain (green); glycine/serine linker (grey); and signal sequence (red). (**b**) GTPase rates of SRP–SR complexes determined in multiple turnover reactions. Curves are shown for SRP containing fl-SRP54 (blue) or fl-SRP54-ss (red), and fl-SRP54-ss in the absence of SRP RNA-SRP19 (black). (**c**) Representative Biacore sensorgrams showing the stabilization of the early SRP–SR complex by the signal sequence. Immobilized SR was probed in the absence of GTP with SRP containing fl-SRP54 (1 μM, blue, and 0.5 μM, light blue) or fl-SRP54-ss (1 μM, red, and 0.5 μM, light red).

The amino-acid sequence C-terminal to αM5 in the M domain varies between species in length and composition, but is found in all SRP54 proteins^21^. On the basis of cross-linking^22^ and cryoelectron microscopy studies^14^, the C-terminal residues are suggested to play a role in signal sequence binding. These residues, which are deleted in most SRP54 constructs used for crystallization, are flexible in the crystal structure of the free *Pyrococcus furiosus* SRP54 (ref. 23). The SRP54 proteins in the crystal structures of the SRP54-signal sequence fusions (SRP54-ss)^5^,^6^ also lack the residues located C-terminal to the αM5. We hypothesized that the reported dimerization of SRP54-ss^5^,^6^ is caused by the absence of the C-terminal aliphatic sequence. To verify this, we cloned and expressed the full-length (fl) SRP54 protein from *M. jannaschii* (aa1-451) fused to a strongly hydrophobic, idealized signal sequence comprising 14 leucine and alanine residues^6^,^24^ (Fig. 1a). The SRP54 C-terminus and the signal sequence were separated in the protein construct by a 9-aa long glycine/serine linker to allow the signal sequence to properly bind to the M domain. The fl-SRP54-ss protein was identical to the previously reported SRP54-ss protein^6^ except for the inclusion of the 20 C-terminal amino acids of *M. jannaschii* SRP54. Size-exclusion chromatography demonstrated that fl-SRP54-ss, but not SRP54-ss^6^, was monomeric in solution (Supplementary Fig. 2).

The presence of a signal sequence accelerates SRP–SR interaction and consequently produces an apparent stimulatory effect on the GTPase reaction of the SRP–SR complex^6^,^16^,^24^,^25^,^26^. To test functionality of fl-SRP54-ss we reconstituted *M. jannaschii* SRP core complexes comprising SRP54, SRP19 and SRP RNA. As expected, we observed that SRP complexes containing fl-SRP54-ss stimulated GTP hydrolysis at a rate ∼20 times faster than SRP complexes containing SRP54-ss (Fig. 1b). Moreover, using surface plasmon resonance (Biacore) we found that the signal sequence stabilized the formation of the GTP-independent early NG domain–SR intermediate (Fig. 1c) as is also seen in the *E. coli* SRP^16^,^24^. Thus, the biochemical data demonstrated that the signal sequence fused to fl-SRP54 mimicked a functional signal sequence and that the key functions of SRP54 were unaffected by the linker region. As such, the fl-SRP54-ss provided a simple and relevant model system for structural analysis of signal sequence recognition.

### The asymmetric unit contains two M domain conformations

Crystals of the *M. jannaschii* ternary complex of fl-SRP54-ss, SRP19 and SRP RNA only diffracted to low resolution. Previous structural studies of SRP54 had identified a hinge region (LGMGD aa294-298) positioned between the NG domain and GM linker, which allows for some degree of NG domain movements^10^,^12^. To improve crystal quality by reducing conformational heterogeneity, we cloned and expressed an identical fl-SRP54-ss construct but without the N-terminal NG domain. Size-exclusion chromatography confirmed that this GM linker-M domain-signal sequence fusion (aa303-475), referred to as SRP54M-ss hereafter, was monomeric in solution.

**Figure 2:**
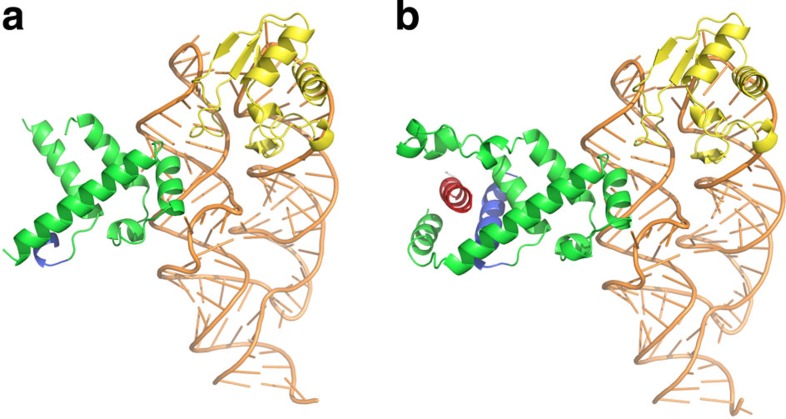
Structure of the *M. jannaschii* SRP54M-ss–SRP19–SRP RNA complex. The two complexes in the asymmetric unit are shown as ribbon representations: (**a**) the unbound form (complex A) and (**b**) the signal sequence bound form (complex B). The colour code is as follows: GM linker (blue), M domain (green), signal sequence (red), SRP19 (yellow) and SRP RNA (orange).

The optimized crystals of the complex between SRP54M-ss, SRP19 and a 96-nucleotide SRP RNA (G142–G237 of *M. jannaschii* SRP RNA) diffracted to 2.9 Å resolution. These crystals belonged to space group P22_1_2_1_ with two complexes in the asymmetric unit. The structure was solved by molecular replacement (Table 1). In the complex structure, SRP19 binds to the tetraloop regions of RNA helices 6 and 8, and the SRP54 M domain binds to the symmetric and asymmetric loops in helix 8, as previously reported^6^,^9^. The two complexes in the asymmetric unit, referred to here as complexes A and B, both show well-defined electron density for the RNA, SRP19 and for residues in αM1 to αM5—excluding the finger loop. These residues form the M domain core and contain the RNA binding motif. There are no significant differences for the RNA and SRP19 in complexes A and B. The structures of the M domain cores are also essentially the same. Indeed, this part of the M domain is rather rigid as evidenced by the relatively low B-factors. However, there is a striking difference in the electron density maps of complexes A and B for the GM linker, finger loop and C-terminal sequence (Supplementary Fig. 3). In complex A, these elements are highly flexible and could not be modelled, whereas in complex B they are ordered (Fig. 2). The conformation depends on whether the SRP complex has a signal sequence bound or not.

### Unbound and signal sequence bound conformations of SRP

In what we refer to as complex A, the signal sequence is not bound. Concurrently, residues in the GM linker (M303-I318), finger loop (K346-H364) and C-terminal sequence (K431-G451) are structurally undefined (Fig. 2a). In this unbound form the M domain contains five helices (αM1–αM5), whose structures and spatial arrangement are identical to those in complex B and previous *M. jannaschii* SRP structures^6^,^9^. The surface of complex A has only a shallow and short hydrophobic groove composed mainly of nonpolar residues from αM1 (L328–M341) and the C-terminal part of αM5 (T419–K429).

**Figure 3:**
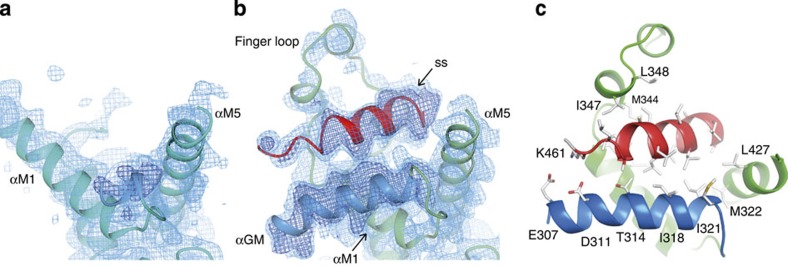
Conformational changes in the M domain induced by signal sequence binding. The electron density map is shown for the signal sequence unbound (**a**) and bound (**b**) forms. To avoid model bias, the signal sequence and GM linker were excluded from the coordinate file subjected to refinement before map calculations. Light blue mesh is 2mFo—DFc density contoured at 0.5 *σ*. Dark blue mesh is mFo—DFc density at+2.5 σ (there is no density at—2.5 *σ* in this area). The refined models of chain C and D are shown as green and cyan ribbons, respectively; the signal sequence in red ribbon; and the GM linker in blue ribbon. (**c**) Interactions at the signal sequence binding site in complex B. Conserved hydrophobic residues (shown as sticks) of the GM linker are positioned on one side of the helix with their side chains directed towards the signal sequence. Also shown is the positively charged lysine residue K461 at the N-terminus of the signal sequence. This residue is located in the vicinity of two conserved acidic residues in the GM linker. The αM6 helix was removed from figures (**b**) and (**c**) for clarity.

**Figure 4:**
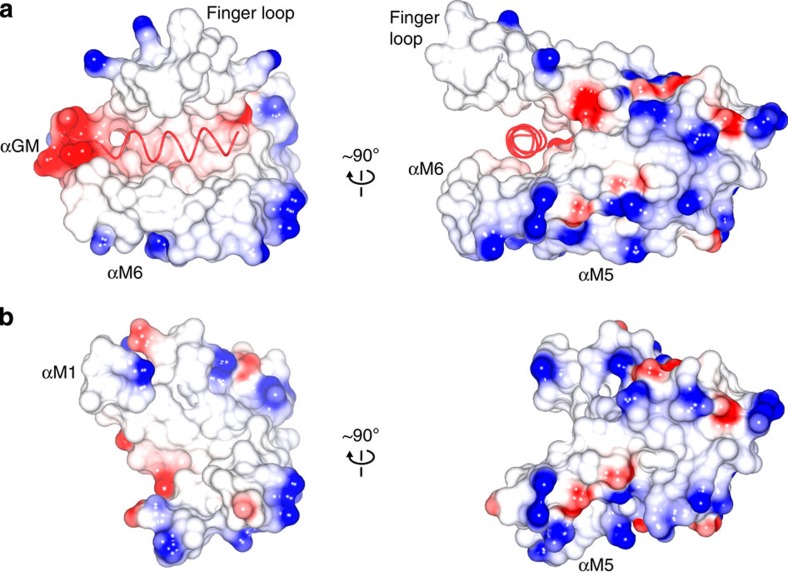
A deep hydrophobic groove is formed upon signal sequence binding. Electrostatic surface representation of the signal sequence bound (**a**) and unbound (**b**) conformations of the *M. jannaschii* M domain. Both structures are shown in the same orientations. The signal sequence is shown as a red worm.

In complex B, the signal sequence forms a 4-turn α-helical structure, deeply embedded into a groove formed by the GM linker, αM1, finger loop, αM5 and the C-terminal sequence (Fig. 2b). The GM linker adopts an α-helical structure (αGM, A306-M322) that is oriented with respect to the signal sequence helix with a crossing angle of ∼30° (Fig. 3). The αGM linker, together with the perpendicular oriented C-terminal part of αM5, compose the bottom of the groove. The αM1 and the finger loop (G342-L365) form one side of the groove. The opposite side is formed by the 20 C-terminal amino acids in the M domain (K431-G451). These residues form a 3-turn α-helical structure (αM6, P438-L446) that runs antiparallel to the signal sequence. Helices αM5 and αM6 are connected by a 90° bend at residue G430. Altogether, these five structural elements—αGM, αM1, finger loop, αM5 and αM6—create a hydrophobic groove about 20 Å deep and 25 Å long, exposing ∼1,500 Å^2^ of surface area (Fig. 4). The αM6 has almost no contact with αGM, and αGM on its part makes relatively few interactions with αM1. These weak inter-helical interactions suggest that the signal sequence-binding surface is rather flexible and allows for a high degree of structural plasticity: The side chains of the binding site residues have generally weaker electron density and higher B-factors than for the rest of the M domain.

Within the binding groove, nonpolar residues form a continuous hydrophobic surface. The hydrophobic character of the groove is conserved in M domains from archaea and bacteria, but the sequence identity is not (Supplementary Fig. 4). Conserved hydrophobic residues lining the groove include: T314, I318, I321 and M322 in αGM; I338 and M341 in αM1; M344, I347, L348, M350, I351 and L362 in the finger loop; T420, A423, I424 and L427 in αM5; and L439, I442 and M443 in αM6. Of these residues, 16 are involved in extensive hydrophobic interactions with the signal sequence. In fact, all of the 14 amino acids in the signal sequence make van der Waal contacts with the hydrophobic groove residues. In total, the binding groove buries 65% of the signal sequence surface area, which corresponds to 980 Å^2^. To this, 27% is contributed by the finger loop, 24% by αGM, 18% by αM1, 17% by αM6, and 14% by αM5.

The finger loop could not be traced in the previous structure of the *M. jannaschii* SRP54-ss dimer^6^, probably because of SRP54 dimerization. Here in our monomeric SRP54M-ss structure, the complete finger loop can be modelled into the electron density. The short helix αMF (P359-L365) in the C-terminal part of the finger loop is well ordered. The remaining 17 finger loop residues (G342-M358), between αM1 and αMF, adopt a traceable, but flexible, extended loop structure with two short helical turns. We conclude that this region does not adopt a rigid conformation upon signal sequence binding.

Whereas polar and charged residues are almost completely excluded from the binding groove, a striking negatively charged surface patch is located at the ‘exit' of the hydrophobic groove in the N-terminus of αGM (Fig. 4). Here conserved acidic residues (E307, D311) are favourably located to interact with positively charged residues in the N-terminus of signal sequences via electrostatic interactions.

## Discussion

In this study, we report the crystal structure of the complex of SRP RNA, SRP19, and a fl-SRP54 M domain-signal sequence fusion from *M. jannaschii*. The crystal contained two copies of the SRP complex in the asymmetric unit. Interestingly, the two M domains in the respective SRP complex adopted significantly different configurations, which represented the signal sequence unbound (complex A) and bound (complex B) forms of the M domain. This crystal packing is best explained by crystal packing interactions unique to complex A that selectively pack only unbound particles into the crystal lattice at this site. This provided us with a unique opportunity to directly compare the structures of unbound and signal sequence bound forms of the fl-SRP54 M domain.

In its unbound form (complex A), the M domain is structurally defined only by its core elements, that is, αM1-αM5, excluding the finger loop. The M domain core is rigid and unchanged from the ones present in complex B and in previously determined *M. jannaschii* SRP structures^6^,^9^. In addition to the finger loop, the GM linker and the C-terminal residues are also disordered, which confirmed that the signal sequence binding site is only partly preformed^9^. As seen in complex B, signal sequence binding triggered a concerted folding of the GM linker, the finger loop, and the C-terminus in such a way that they, together with αM1 and αM5, created a large U-shaped continuous hydrophobic surface that covers three sides of the bound signal sequence (Fig. 4). This induced-fit binding is biologically meaningful because the inherent flexibility of recognition elements, coupled with induced folding, can provide a high degree of structural adaptability. This should facilitate the specific recognition of highly diverse signal sequences. The plasticity of the signal sequence binding groove is further supported by relative few interactions between the recognition elements. Because SRP function is evolutionarily conserved, we expect that this recognition mechanism applies to SRP from other domains of life. In support, biochemical and structural data of ligand-free SRP54 from different species indicate that the GM linker, finger loop and C-terminus are flexible, or captured in variable conformations by crystal contacts^7^,^8^,^10^,^23^. In complex B, the structures of the GM linker, the finger loop and the αM6 are not supported by crystal contacts.

Only limited structural data are available for the SRP54 C-terminus and its role in signal sequence binding has not been elucidated. Here we show that the C-terminus in *M. jannaschii* assumes the shape of an α-helix (αM6) thereby shielding the signal sequence from solvent. *M. jannaschii* SRP54-ss, which is lacking the C-terminus, forms monomers and dimers in a roughly 1:1 ratio in solution^6^. Assembled into SRP complexes, the monomeric form does not accelerate SR interaction or stimulate GTP hydrolysis, showing that the C-terminus is required for SRP function (Supplementary Fig. 5a,b). However, the dimeric form shows strongly enhanced activity. Intriguingly, in the structure of the dimer^6^ the symmetry-related signal sequence helix is positioned similarly to the C-terminus in SRP54M-ss. This confines the signal sequence within the binding site and thus explains its rescued activity (Supplementary Fig. 5c).

Superimposition of the SRP54-ss and SRPM-ss structures shows a virtually identical orientation of the bound signal sequence relative to αGM and the M domain core. Moreover, the same contacts are made by the signal sequence with the hydrophobic residues in αGM and αM1. The importance of some of these residues for SRP function is confirmed by mutational analysis^6^. The signal sequence binding site observed in *M. jannaschii*, however, differs from those seen in the crystal structure of the *S. solfataricus* SRP54-ss^5^ and the cryoelectron microscopy structures of the *E. coli* SRP–RNC complex^12^,^14^. In these structures, the signal sequence is oriented roughly antiparallel to αM5 (Supplementary Fig. 1). Comparison of the *M. jannaschii* and *S. solfataricus* binding sites shows that some hydrophobic contacts are conserved, for example, I338 (L339 *in S. solfataricus*) in αM1, and T420 (M424) and I424 (L428) in αM5. The different binding sites could represent discrete binding modes that occur during the recognition event. This interpretation agrees well with recent data demonstrating that SRP binds to RNCs with exposed signal sequences in multiple interconverting conformations^27^. Docking of complex B onto the ribosome of the *E. coli* SRP–RNC complex^12^,^14^ shows a good fit without steric clashes (Supplementary Fig. 6).

Successive events during the SRP cycle require a rearrangement of the relative position of the NG and M domains^11^,^12^,^14^,^17^,^18^,^28^. Here we show that on signal sequence binding, the GM linker folds into an ordered α-helix (αGM). This αGM is an integral structural component of the complete binding site and makes intimate contact with the signal sequence (Fig. 3c). The αGM follows the path of the αGM helix in the dimer structure^6^. In the latter structure, the NG domain has rotated 180° with respect to its position in the free form, which brings the GTPase domain in proximity to the tip of helix 8 (refs 6, 9). We conclude that signal sequence-induced folding of αGM is the key event that directly links ligand binding to NG domain repositioning. We speculate that this folding event enforces an αGM orientation that predisposes the NG domain for interaction with SR to form the early NG domain–SR complex at helix 8 tetraloop (Fig. 1c and ref. 6). GTP hydrolysis and signal sequence release take place after the closed NG domain–SR complex has repositioned to helix 5 (refs 11, 17, 18). In the signal sequence release state, αGM is directed away from the M domain^11^. This αGM orientation is not compatible with signal sequence binding as seen in complex B. It is therefore conceivable that NG domain–SR repositioning to helix 5 promotes the disassembly of the binding groove and thus exposure of the signal sequence for signal sequence release.

The coupling of ligand binding and folding of the recognition region in the M domain (including the critical GM linker) provides a simple and efficient mechanism to communicate signal sequence recognition in the M domain to the NG domain. Moreover, we propose that signal sequence recognition via a disorder-to-order transition of multiple structural elements facilitates specific recognition of widely diverse signal sequences. Future structural studies with signal sequences of different composition and length will be necessary to gain a more comprehensive view of how promiscuous binding by SRP is achieved.

## Methods

### Protein and RNA production, complex assembly and crystallization

The *srp19* gene from *M. jannaschii* was cloned into the pET-3a vector (Novagen) and expressed in JM109(DE3) cells (Promega). The SRP19 protein was purified by heat treatment followed by sequential column chromatography on Heparin Sepharose, Mono S, and Superdex 75 (GE Healthcare). The DNA sequence for *M. jannaschii* fl-SRP54, fl-SRP54-ss, SRP54-ss and SRP54M-ss was cloned into the pNZ8048 vector and expressed in *Lactococcus lactis*. The SRP54 proteins were purified by heat treatment followed by sequential column chromatography on Heparin Sepharose, Mono S and Superdex 200 (GE Healthcare). The *M. jannaschii* SRP RNA was transcribed *in vitro* using T7 RNA polymerase and purified by denaturing gel electrophoresis. Before complex formation, the RNA was annealed in water by denaturation at 75 °C followed by snap cooling on ice. The annealed RNA was purified on Mono Q (GE Healthcare) and dialyzed against 10 mM Tris-HCl (pH 7.5), 5 mM MgCl_2_. The protein–RNA complex was reconstituted in a buffer containing 10 mM Tris-HCl (pH 7.5), 250 mM KCl, 5 mM MgCl_2_, 1% (v/v) ß-mercaptoethanol. Binding reactions were incubated for 15 min at room temperature after addition of SRP19, and for 1 h at 37 °C after addition of SRP54. After this, the complex was purified on a Mono Q column and dialyzed against 10 mM Tris-HCl (pH 7.5), 250 mM KCl, 5 mM MgCl_2_. The complex (3 mg/ml) was crystallized by the hanging-drop vapor-diffusion technique at 18 °C. Crystals (0.15 × 0.15 × 0.05 mm^3^) grew in 7 days when the complex solution was mixed with an equal volume of mother liquor containing 35% 2-Methyl-2,4-pentanediol, 200 mM NaCl, 80 mM MgCl_2_, 100 mM sodium acetate (pH 5.0).

### Data collection, phasing and refinement

Crystals were cryocooled directly from mother liquor and diffraction data were collected at the European Synchrotron Radiation Facility beamline ID23-1 using X-ray radiation with *λ*=0.984 Å at 100 K. Data were processed and scaled using XDS^29^ and SCALA from the CCP4 suite^30^. Statistics from data collections are listed in Table 1. Crystals of the *M. jannaschii* SRP54M-ss, SRP19 and SRP RNA complex belonged to space group P22_1_2_1_and contained two molecules in the asymmetric unit. The structure was solved by molecular replacement using the programme PHASER from the PHENIX suite^31^, and the SRP54-ss complex (pdb code 3NDB)^6^ as a search model. The electron density map was well defined in complex A and B for SRPRNA, SRP19 and the M domain core. In complex B the electron density also defined the structures of the signal sequence, GM linker, finger loop and αM6. A distinctive feature of the signal sequence, and the residues constituting its binding site, is the weak electron density for their side chains relative to those in the rest of the molecule. The structures were built and refined using the programs COOT^32^ and PHENIX REFINE. Structures were superimposed in COOT using SSM^33^. Refinement statistics of the structures are given in Table 1. The model has no outliers in the Ramachandran plot and the clashscore is 1. Representative electron density is shown in Supplementary Fig. 7 in stereo. The accessible surface area of the signal sequence binding groove was calculated using the CASTp server^34^. Figures 2 and 3c and Supplementary Information Supplementary Figs 1, 5c and 6 were prepared with PyMOL^35^; Figs 3a,b and 4 and Supplementary Information Supplementary Fig. 3 were prepared with CCP4mg^36^.

### GTPase assay

One micromolar of *M. jannaschii* SRP RNA (nucleotides G123–C258, which include the helix 5 region required for GTPase activation^11^,^17^), 1.5 μM of SRP19 and 0.5 μM of SRP54 were assembled in binding buffer containing 10 mM Tris-Cl (pH 7.5), 150 mM KCl, 5 mM MgCl_2_, and 1% (v/v) ß-mercaptoethanol to form 0.5 μM of *M. jannaschii* SRP. *M. jannaschii* SR(aa93-408) was added to the SRP complex to final concentrations of 0, 1, 2, 5, 10 and 25 μM. GTP hydrolysis reactions, carried out at 25 °C, were started by adding 100 μM GTP with trace amounts of γ-^32^P-GTP (PerkinElmer). At different time points, 0.5 μl aliquots of the reactions were spotted on a polyethyleneimine–cellulose thin-layer chromatography (PEI-TLC) plate (Merck). Inorganic phosphate (P_i_) and GTP were separated by chromatography in 0.75 M KH_2_PO_4_ and quantified with a Phosphorimager (Molecular Dynamics). Rates of GTP hydrolysis were averaged from three independent experiments and fitted to a single exponential model.

### Surface plasmon resonance

Sensorgrams were recorded using a Biacore 3,000 instrument (GE Healthcare). An anti-gluthathion-S-transferase (GST) monoclonal antibody was immobilized on a CM-5 chip (GE Healthcare) by amine coupling according to the manufacturer's instructions. Binding surfaces were subsequently generated by the application of equimolar amounts of GST-only as reference, or the N-terminally GST-tagged *M. jannaschii* SR(aa93-408). Interaction experiments were performed in running buffer containing 10 mM Tris-Cl (pH 7.5), 150 mM KCl, 5 mM MgCl_2_ at 37 °C. Purified *M. jannaschii* SRP complexes in running buffer were injected at 0.5 and 1 μM concentrations in the absence of GTP and a flow rate of 5 μl min^−1^. Sensorgrams were corrected for non-specific interaction with the GST-only surface and by double referencing.

## Additional information

**Accession codes**: Coordinates and structure factors have been deposited with the Protein Data Bank under the accession code 4XCO.

**How to cite this article:** Hainzl, T. *et al*. Signal sequence induced conformational changes in the signal recognition particle. *Nat. Commun.* 6:7163 doi: 10.1038/ncomms8163 (2015).

## Supplementary Material

Supplementary Information Supplementary Figures 1-7

## Figures and Tables

**Table 1:** Data collection and refinement statistics.

	SRP54M-ss
*Data collection*	
Space group	P22_1_2_1_
Cell dimensions	
*a*, *b*, *c* (Å)	91.23,112.97,121.21
α, β, γ (°)	90.0, 90.0, 90.0
Resolution (Å)	48.0–2.90(3.08–2.90) *
*R*_merge_	0.073 (0.899)
*I* / σ*I*	13.7 (1.9)
Completeness (%)	99.7 (99.7)
Redundancy	6.5 (6.7)
	
*Refinement*	
Resolution (Å)	48.0–2.90
No. reflections	28330 (2768)
*R*_work_ / *R*_free_	0.209 (0.318)/0.258(0.373)
No. atoms	
Protein	3390
RNA	4124
Ligand/ion	15
Water	70
*B*-factors	
Protein	104.9
RNA	91.7
Ligand/ion	89.9
Water	85.7
R.m.s. deviations	
Bond lengths (Å)	0.002
Bond angles (°)	0.520

^*^One crystal was used for data collection. Values in parentheses are for highest-resolution shell.
