# Supplementary material for: Signal-sequence induced conformational changes in the signal recognition particle
Source: Nat Commun. 2015 Jun 8;6:7163. doi: 10.1038/ncomms8163 (PMC4468861; doi:10.1038/ncomms8163)
Supplement: Supplementary Information — Supplementary Figures 1-7 [file ncomms8163-s1.pdf]

Supplementary Information for:

Signal-sequence induced conformational changes in the signal recognition particle

Tobias Hainzl & A. Elisabeth Sauer-Eriksson

Department of Chemistry, Umeå University, SE-901 87 Umeå, Sweden

Supplementary Figures 1-7

Supplementary References

Supplementary Figure 1

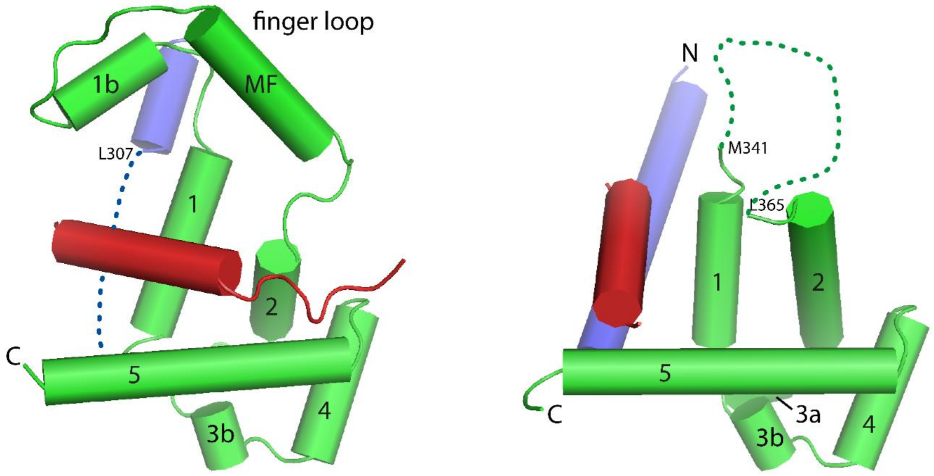

Supplementary Figure 1. The crystal structures of SRP54-signal sequence complexes show different orientations of the signal sequence. Orientation of the signal sequence in the M domain of the *S. solfataricus* SRP54-signal sequence fusion dimer (pdb code 3KL4[1], left) and the *M. jannaschii* SRP54-signal sequence fusion dimer (pdb code 3NDB[2], right). Cartoon representations with the GM linker in blue, the M domain in green and the signal sequence in red. Blue and green dotted lines represent the flexible GM linker and finger loop residues. The α-helices in the M domain are labeled 1 through 5.

Supplementary Figure 2

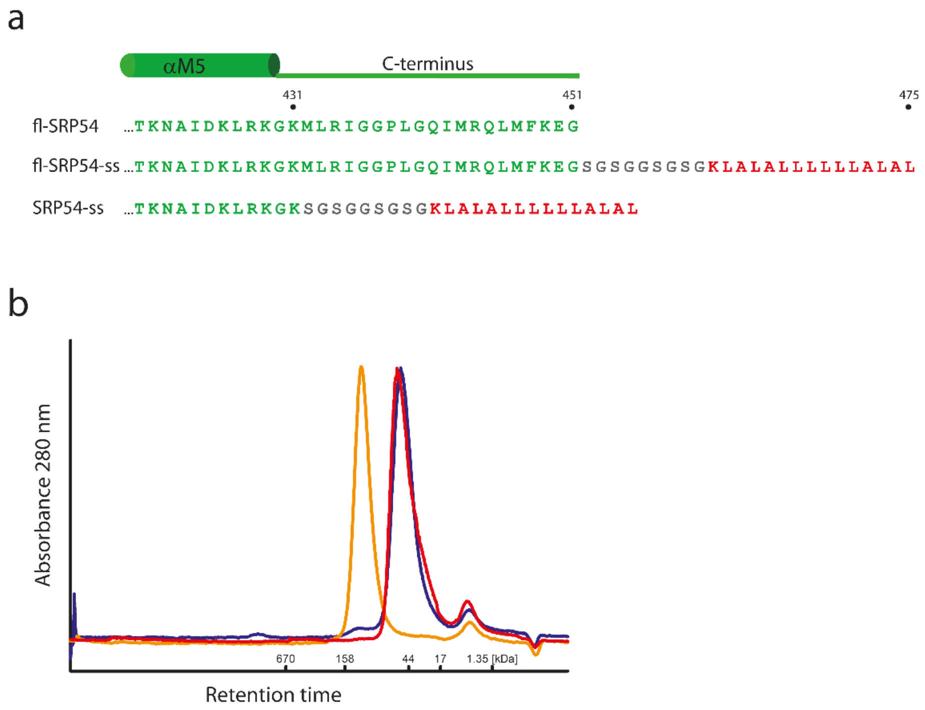

Supplementary Figure 2. The fl-SRP54-ss is a monomer in solution. (**a**) C-terminal amino acid sequences of *M. jannaschii* SRP54 constructs used in this study. The color code is as follows: M domain (green), glycine/serine linker (gray) and signal-sequence (red). (**b**) Size exclusion chromatography of *M. jannaschii* SRP54 proteins. The elution profiles from a Superdex-S200 column (GE Healthcare) in a buffer containing 10 mM Tris-Cl (pH 7.5), 250 mM KCl and 5 mM $MgCl_2$ are shown for fl-SRP54-ss (red), fl-SRP54 (blue) and SRP54-ss dimer[2] (orange). The elution positions and molecular weights (in kilodalton, kDa) of standard proteins are indicated.

Supplementary Figure 3

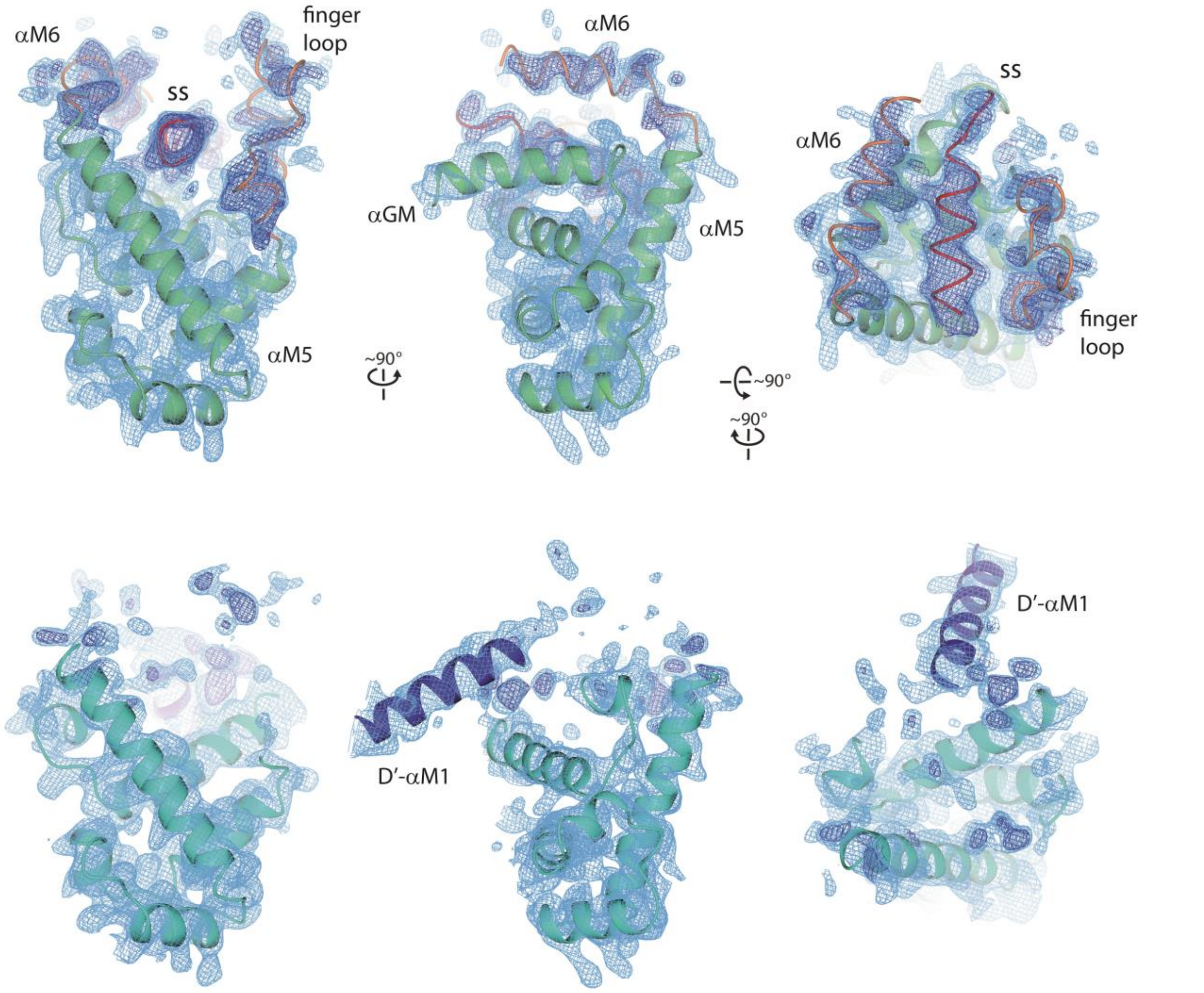

Supplementary Figure 3. Quality of the electron density prior to placement of the signal sequence, finger loop, and αM6 helix. The upper panel shows three orientations of complex B, and the lower panel the same orientations for complex A. To avoid model bias, these structural elements were excluded from the coordinate file subjected to refinement before map calculations. Lightblue mesh is 2mFo – DFc density contoured at 0.5 σ. Darkblue mesh is mFo – DFc density at + 2.5 σ (there is no density at – 2.5 σ in this area). For clarity, only mFo – DFc density within a 3 Å radius of the placed structures is displayed. The refined model of chain C and D are shown as green and cyan ribbons, respectively; the finger loop and αM6 are shown as coral worms; and the signal sequence is shown as a red worm. The αM1 helix from a symmetry related complex A is shown as a darkblue ribbon (D′-αM1)

Supplementary Figure 4

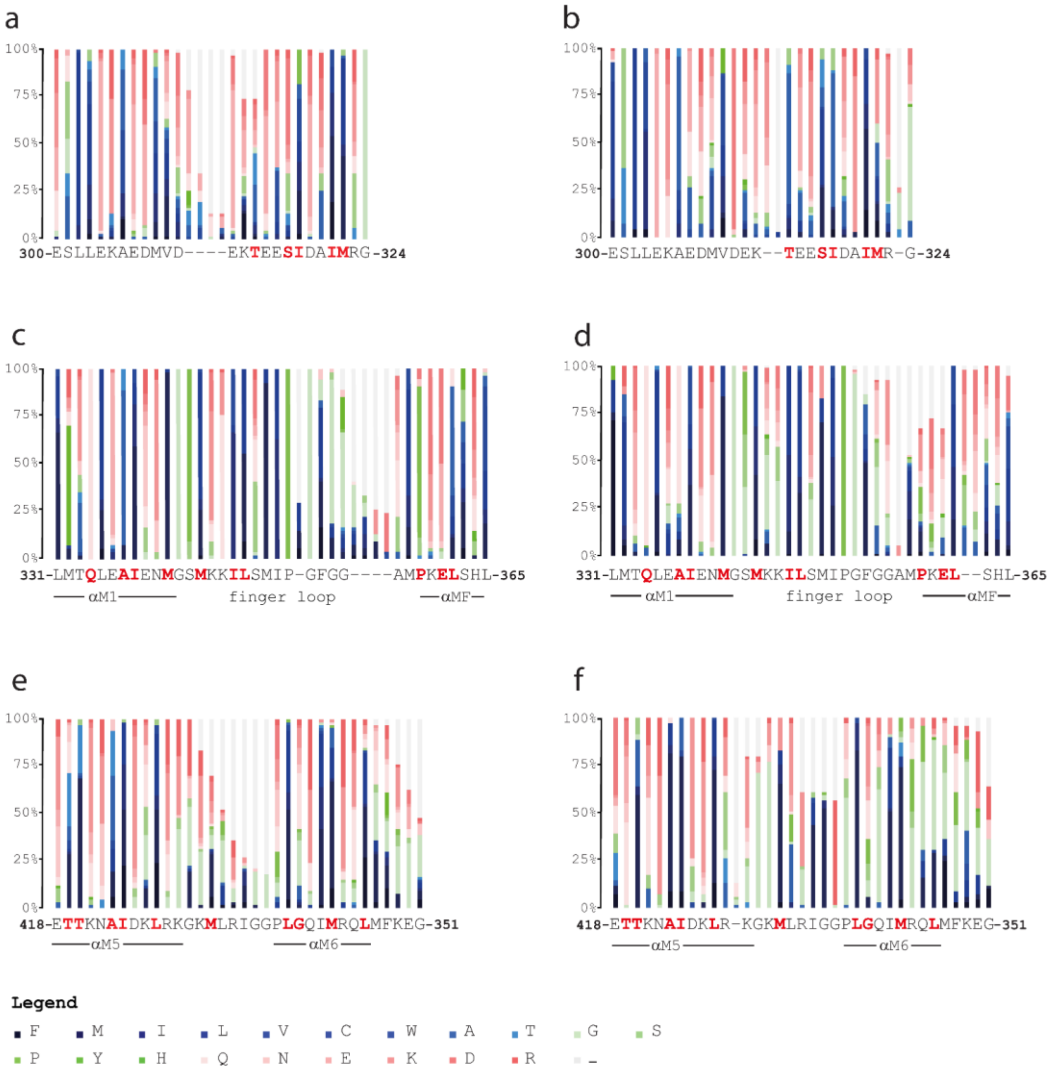

Supplementary Figure 4. The signal-sequence binding groove is lined by conserved hydrophobic residues. Multiple sequence alignments and frequency plots for the structural elements in archaeal and bacterial SRP54 proteins that compose the signal sequence binding site. Alignments of 67 archaeal and 69 bacterial species were generated using Clustal Omega[3]. The percentage of occurrence of each amino acid is plotted. The amino acids, listed in the legend, are colored according to their hydrophobic and hydrophilic character from blue colors (hydrophobic) to green and red colors (polar and charged). Upper panel: Frequency plots for the GM-linker sequence in (**a**) archaea and (**b**) bacteria. Middle panel: Frequency plots for the αM1 and finger-loop sequences in (**c**) archaea and (**d**) bacteria. Lower panel: Frequency plots for the αM5 (C-terminal part) sequence and SRP54 C-terminus in (**e**) archaea and (**f**) bacteria. The plot in **e** and **f** is truncated at the C-terminal end of *M. jannaschii* SRP54. The sequence of *M. jannaschii* SRP54 is shown below. Residues in the interface with the signal sequence are highlighted in red (determined using PISA[4]).

Supplementary Figure 5

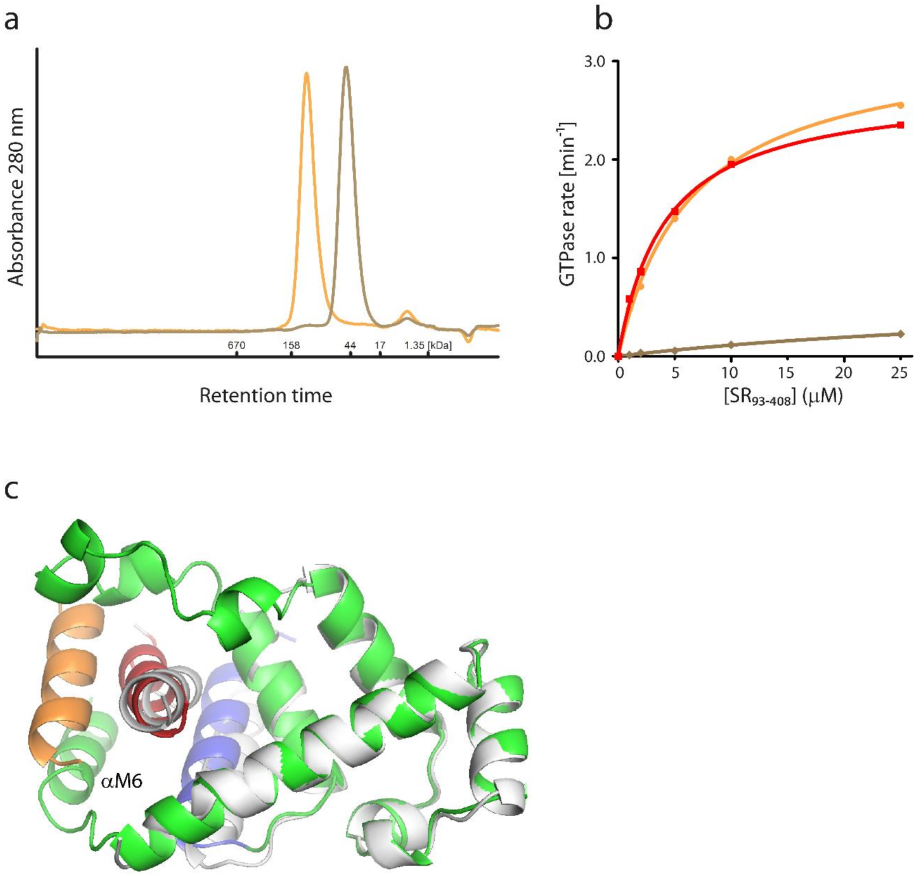

Supplementary Figure 5. The SRP54 C-terminus is essential for SRP function (**a**) Size exclusion chromatography of SRP54-ss dimer and monomer from *M. jannaschii*. The elution profiles from a Superdex-S200 column (GE Healthcare) in a buffer containing 10 mM Tris-Cl (pH 7.5), 250 mM KCl and 5 mM $MgCl_2$ are shown for the dimer (orange) and for the monomer (brown). The elution positions and molecular weights (in kilodalton, kDa) of standard proteins are indicated. (**b**) GTPase rates of SRP-SR complexes determined in multiple turnover reactions. Curves are shown for SRP containing the SRP54-ss dimer (orange), SRP containing the SRP54-ss monomer (brown), and SRP containing the fl-SRP54-ss (red). (**c**) Overlay of the structures of the M domains from one monomer of the SRP54-ss dimer (chain B, pdb code 3NDB[2]) and SRP54M-ss (chain C) based on the superimposition of the M domain cores. Cartoon representation with a color code for SRP54M-ss as follows: GM linker (blue), M domain (green), signal sequence (red). The monomer of the SRP54-ss dimer and bound signal sequence is shown in white with the symmetry-related signal sequence at the dimer interface shown in orange. The αM6 and the symmetry-related signal sequence both shield the signal sequence from solvent and confine the signal sequence within the hydrophobic binding site. The αM6 in SRP54M-ss buries 170 $Å^2$ of the signal-sequence surface area. Similarly, the symmetry-related signal sequence buries 240 $Å^2$ of the signal-sequence surface area in SRP54-ss.

Supplementary Figure 6

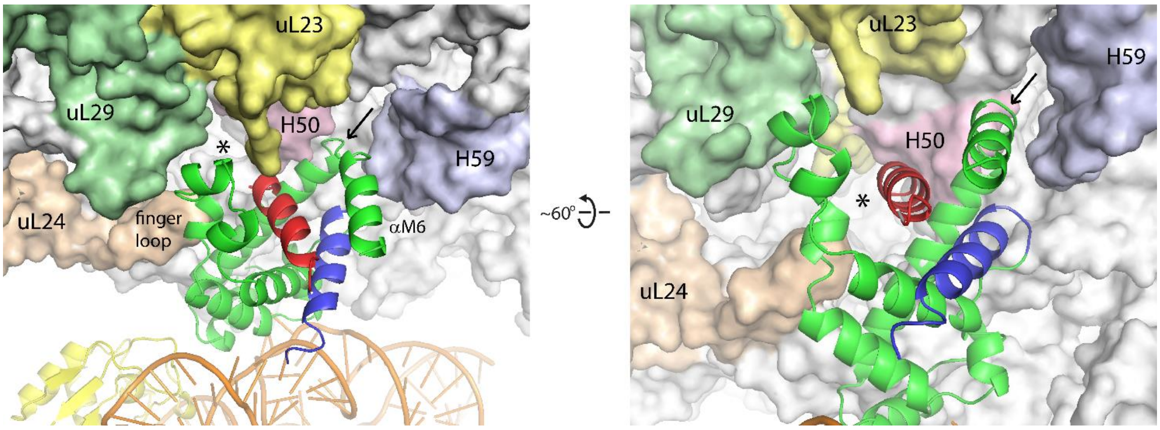

Supplementary Figure 6. Model of complex B on the ribosome. The model is based on the superposition of the respective M domain cores of complex B and *E. coli* SRP-RNC complex (pdb code 2J28[5]). Complex B is shown as a ribbon representation whereas the 50 S ribosomal subunit is shown as a surface representation. Color code is as follows: SRP RNA (orange), SRP19 (yellow), SRP M domain (green), GM linker (blue), signal sequence (red), ribosomal protein uL23 (yellow), uL24 (orange), uL29 (green), ribosomal RNA helix 50 (pink) and helix 59 (blue). Asterisk denotes the ribosomal nascent chain exit site. Additional stabilization to the M domain conformation may be provided by contacts with ribosomal components. In particular, conserved basic residues in the region connecting αM5 and αM6[6] (indicated by an arrow) are ideally located for interaction with RNA helices 50 and 59.

Supplementary Figure 7

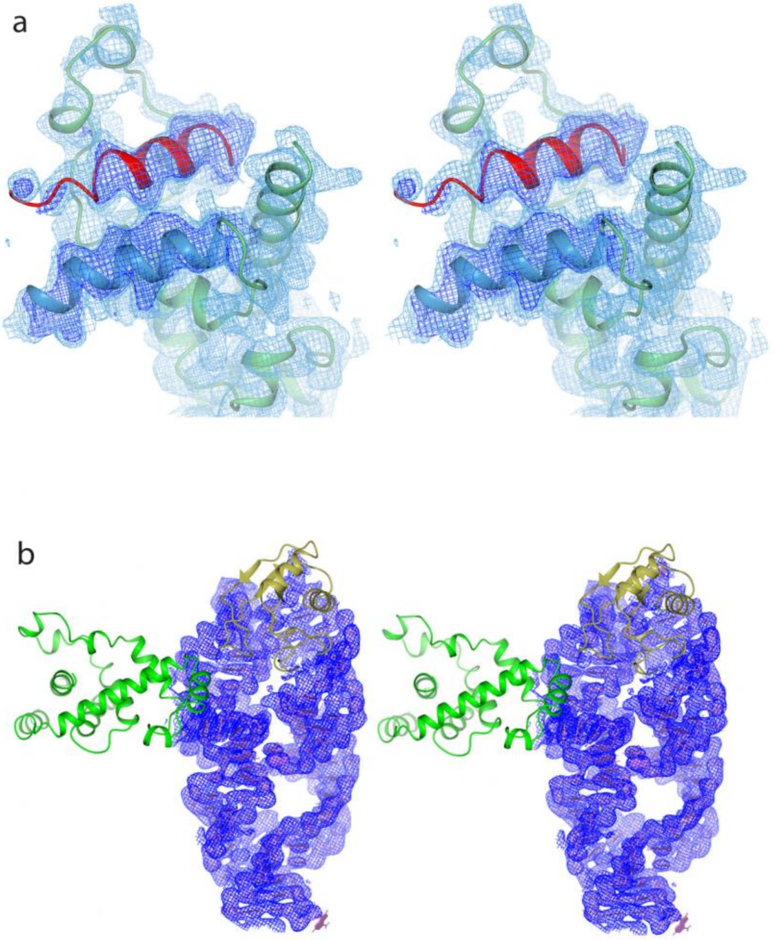

Supplementary Figure 7. Stereo image of the electron density (**a**) Quality of the electron density at the signal sequence binding site in complex B. To avoid model bias, the signal sequence and GM linker were excluded from the coordinate file subjected to refinement before map calculations. Lightblue mesh is 2mFo – DFc density contoured at 0.5 σ. Darkblue mesh is mFo – DFc density at + 2.5 σ (there is no density at – 2.5 σ in this area). M domain, signal sequence, and GM linker are shown as green, red, and blue ribbon, respectively. The αM6 helix was removed from the figure for clarity. (**b**) Quality of the electron density over the RNA chain only in complex B. Blue mesh is the 2mFo – DFc density contoured at 1.0 σ. M domain, SRP19, and RNA are shown as green, yellow and pink ribbon, respectively.

Supplementary References

1 Janda, C. Y. *et al.* Recognition of a signal peptide by the signal recognition particle. *Nature* **465**, 507-510, doi:10.1038/nature08870 (2010).

2 Hainzl, T., Huang, S., Merilainen, G., Brannstrom, K. & Sauer-Eriksson, A. E. Structural basis of signal-sequence recognition by the signal recognition particle. *Nature structural & molecular biology* **18**, 389-391, doi:10.1038/nsmb.1994 (2011).

3 Sievers, F. *et al.* Fast, scalable generation of high-quality protein multiple sequence alignments using Clustal Omega. *Molecular systems biology* **7**, 539, doi:10.1038/msb.2011.75 (2011).

4 Krissinel, E. & Henrick, K. Inference of macromolecular assemblies from crystalline state. *Journal of molecular biology* **372**, 774-797, doi:10.1016/j.jmb.2007.05.022 (2007).

5 Halic, M. *et al.* Following the signal sequence from ribosomal tunnel exit to signal recognition particle. *Nature* **444**, 507-511, doi:10.1038/nature05326 (2006).

6 Andersen, E. S. *et al.* The tmRDB and SRPDB resources. *Nucleic acids research* **34**, D163-168, doi:10.1093/nar/gkj142 (2006).
